# Ultrathin 2D In_2_O_3_ Nanosheets Confining Cu Single Atoms Synergized With Oxygen Vacancies for High Selectivity of Photocatalytic Reduction of CO_2_ to CO

**DOI:** 10.1002/advs.77464

**Published:** 2026-08-30

**Authors:** Yueying Yang, Xuan Xiang, Jiali Ren, Ying Bian, Xinyu Wang, Xiaoyang Lu, Yanjun Xue, Porun Liu, Cheng‐Hao Chuang, Xiaoli Zhang, Jian Tian

**Affiliations:** ^1^ State Key Laboratory of Disaster Prevention and Ecology Protection in Open‐pit Coal Mines Shandong Key Laboratory of Special Epoxy Resin School of Materials Science and Engineering Shandong University of Science and Technology Qingdao China; ^2^ Dongying Vocational College Dongying China; ^3^ School of Environment and Science Gold Coast Campus Griffith University Gold Coast Queensland Australia; ^4^ Department of Physics Tamkang University, New Taipei Tamsui Taiwan; ^5^ Australian Research Council Centre of Excellence for Green Electrochemical Transformation of Carbon Dioxide Applied Chemistry and Environmental Science School of Science RMIT University Melbourne Victoria Australia

**Keywords:** 2D ultrathin, high selectivity, In_2_O_3_, photocatalytic CO_2_ reduction, single atom

## Abstract

In this work, we constructed an ultrathin two‐dimensional (2D) In_2_O_3_ nanosheet (In_2_O_3_‐Ns) structure based on oxygen vacancies (O_V_)‐rich defects and low crystallinity to anchor Cu single atoms (Cu SAs) and synthesized Cu/In_2_O_3_‐Ns photocatalysts. Benefiting from the synergistic strategy of Cu single atoms and In^3+^‐O_V_, CO_2_ molecules were activated, the energy barrier for the formation of the ^*^COOH intermediates was lowered, and the CO yield is increased to 17.24 µmol g^−1^ h^−1^ with near‐unity selectivity (>99%). Moreover, Cu/In_2_O_3_‐Ns photocatalysts demonstrate exceptional stability over extended cycling tests (64 h). In situ spectral characterization and DFT calculations reveal that the engineered two‐dimensional ultrathin In_2_O_3_‐Ns structures demonstrate significant advantages in promoting CO_2_ enrichment and confining key reaction intermediates following the anchoring of Cu single atoms, thereby substantially enhancing the surface coverage of ^*^COOH species. Cu single atoms and In^3+^‐O_V_ sites synergistically promote ^*^CO_2_ adsorption, ^*^COOH formation/desorption, and accelerate the transfer of photoexcited charge carriers between In_2_O_3_ matrix and Cu single atoms. This cooperative mechanism significantly enhances both the photocatalytic CO_2_ reduction activity and CO selectivity. This work provides a novel strategy for constructing precisely tailored active sites in photocatalysts and offers fundamental insights into boosting the efficiency and selectivity of photocatalytic CO_2_ conversion.

## Graphical abstract

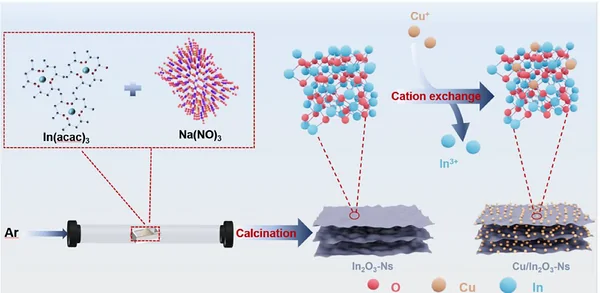

## 1 Introduction

Confronting the dual challenges of global climate change and energy sustainability, the solar‐driven conversion of CO_2_ into value‐added chemical feedstocks and synthetic fuels has emerged as a transformative approach in sustainable chemistry [1]. Among the various products of CO_2_ photoreduction, CO has gained significant attention due to its versatile industrial applications, such as serving as a feedstock for Fischer–Tropsch synthesis and as a reductant in metal smelting [2]. Despite the promising prospects, the practical application of photocatalytic CO_2_ reduction technology still faces significant challenges, with the primary bottleneck being the insufficient intrinsic activity and product selectivity of the catalysts [3, 4]. The fundamental challenges predominantly originate from two aspects. First, the CO_2_ molecule exhibits pronounced chemical inertness and a stable linear molecular geometry, necessitating highly efficient active sites to surmount its substantial activation energy barrier and steer the reaction pathway toward the generation of products with high selectivity [5, 6]. Second, the rapid recombination kinetics of photogenerated electron‐hole pairs severely constrains the overall solar‐to‐chemical energy conversion efficiency [7, 8]. Consequently, the concurrent optimization of both the microenvironment of the catalyst's active sites and the charge‐carrier dynamics is paramount for enhancing the performance of photocatalytic CO_2_ reduction.

In recent years, single‐atom catalysts (SACs) have demonstrated unique potential in catalyzing the directional conversion of CO_2_, owing to their maximized atomic utilization efficiency and tunable local coordination environments [9, 10]. In contrast to noble metal single atoms such as Ru [5], Pt [11], and Pd [12], which face challenges for large‐scale applications due to resource scarcity and high cost, the earth‐abundant copper (Cu) exhibits a moderate adsorption strength for key intermediates (e.g., ^*^CO, ^*^COOH) in the CO_2_ reduction reaction. This property enables it to effectively activate CO_2_ without causing excessive adsorption that would hinder intermediate desorption, thereby favoring a self‐sustaining reaction process and making it a more promising candidate for research [13, 14]. However, the catalytic performance of single atoms is critically dependent on their local coordination environment and the properties of the support [15]. Anchoring single atoms onto a support rich in oxygen vacancies (O_V_) is considered an effective strategy for tuning their electronic states and enabling synergistic catalysis [16, 17].

O_V_ in metal oxides have attracted considerable attention due to their ability to effectively modulate electronic properties and generate adsorption sites [18, 19]. The vacant sites are typically flanked by cations exhibiting high symmetry. However, the uniform electron distribution resulting from this high cation symmetry confines electrons at the O_V_ site, thereby diminishing electron transfer efficiency. To circumvent the limitations of conventional symmetric oxygen vacancies in electronic modulation, researchers have proposed the concept of asymmetric oxygen vacancies (labelled M_1_‐O_V_‐M_2_) [20, 21]. This strategy aims to break the chemical homogeneity of the metal centers on either side of the vacancy by engineering different coordination numbers or valence states, thereby creating a localized asymmetric charge distribution. In this configuration, O_V_ effectively regulates the electronic structure of the active site through its different coordination states and dangling bond properties, while the chemical heterogeneity between the metals enhances the polarization of the reacting molecules, which further facilitates the adsorption and activation processes of small molecules such as CO_2_ and H_2_O [22]. O_V_ not only act as activation sites for CO_2_ molecules to lower their activation energy barriers, but also act as defective states to inhibit the complexation of photogenerated electron‐hole pairs [23, 24, 25]. The construction of two‐dimensional ultrathin metal oxide nanosheet structures featuring high specific surface area and abundant surface oxygen vacancies, which possess high specific surface areas and abundant surface defects, can not only expose more active sites and promote reactant adsorption but may also significantly modulate the coverage and residence time of intermediates through confinement effects, thereby intervening in the reaction pathway from both electron transfer and kinetic perspectives. Integrating atomic‐scale active site design with two‐dimensional carrier engineering offers an opportunity to gain deeper insights into how multiscale structures regulate reaction mechanisms.

Despite the significant advantages of SACs, O_V_, and two‐dimensional ultrathin materials in photocatalytic CO_2_ reduction, several critical bottlenecks remain. First, maintaining the atomic dispersion of metals generally requires stringent constraints on metal loading (typically < 3 wt.%), which severely limits the overall catalytic productivity [26, 27]. Achieving high‐loading single atoms without agglomeration remains a formidable challenge. Second, although isolated O_V_ facilitate CO_2_ adsorption, they are prone to deactivation during the reaction and lack sufficient tunability to optimally stabilize key intermediates such as ^*^COOH. Finally, the integration of high‐density single atoms and oxygen vacancies into a unified two‐dimensional ultrathin framework to construct stable dual‐site synergistic interfaces is synthetically highly challenging, and the underlying mechanisms remain underexplored [28, 29]. Therefore, there is an urgent need to rationally design robust two‐dimensional catalysts that synergistically combine high‐loading single atoms with abundant oxygen vacancies, thereby transcending the catalytic limitations of single active sites.

Herein, we report the synthesis of ultrathin two‐dimensional In_2_O_3_ nanosheets with low crystallinity and rich O_V_. Subsequently, Cu single atoms were anchored onto the defect‐rich nanosheets through a cation‐exchange strategy, yielding Cu single‐atom and O_V_ co‐modified In_2_O_3_ nanosheets (Cu/In_2_O_3_‐Ns) for highly selective photocatalytic CO_2_ reduction to CO. The confinement effect of 2D ultrathin In_2_O_3_ nanosheets with abundant oxygen vacancies could anchor Cu single atoms at room temperature to form the Cu‐O_V_‐In interface. The strong electronic interaction between Cu single atoms and the In_2_O_3_ matrix induced the formation of charged‐polarized Cu single atoms and In^3+^‐O_V_ active sites. The ultrathin 2D nanosheet structure demonstrated significant advantages in promoting CO_2_ enrichment and restricting key reaction intermediates, thereby greatly enhancing the surface coverage of ^*^COOH. In situ spectral characterization and DFT calculation indicated that the In^3+^‐O_V_ active sites significantly promoted the chemisorption and bending of CO_2_ molecules, greatly reduces their activation energy barrier. Cu single atoms could effectively transfer electrons to CO_2_ molecules activated by In^3+^‐O_V_ sites to generate *COOH and further facilitate their conversion to ^*^CO. The formed Cu‐O_V_‐In interface structure promoted the enrichment of electrons, thereby facilitating the formation of ^*^COOH intermediates. This work offers new insights into enhancing the activity and selectivity of photocatalytic reduction of CO_2_ through single‐atom, vacancy, and 2D ultrathin sheet structure.

## 2 Results and Discussion

**FIGURE 1:**
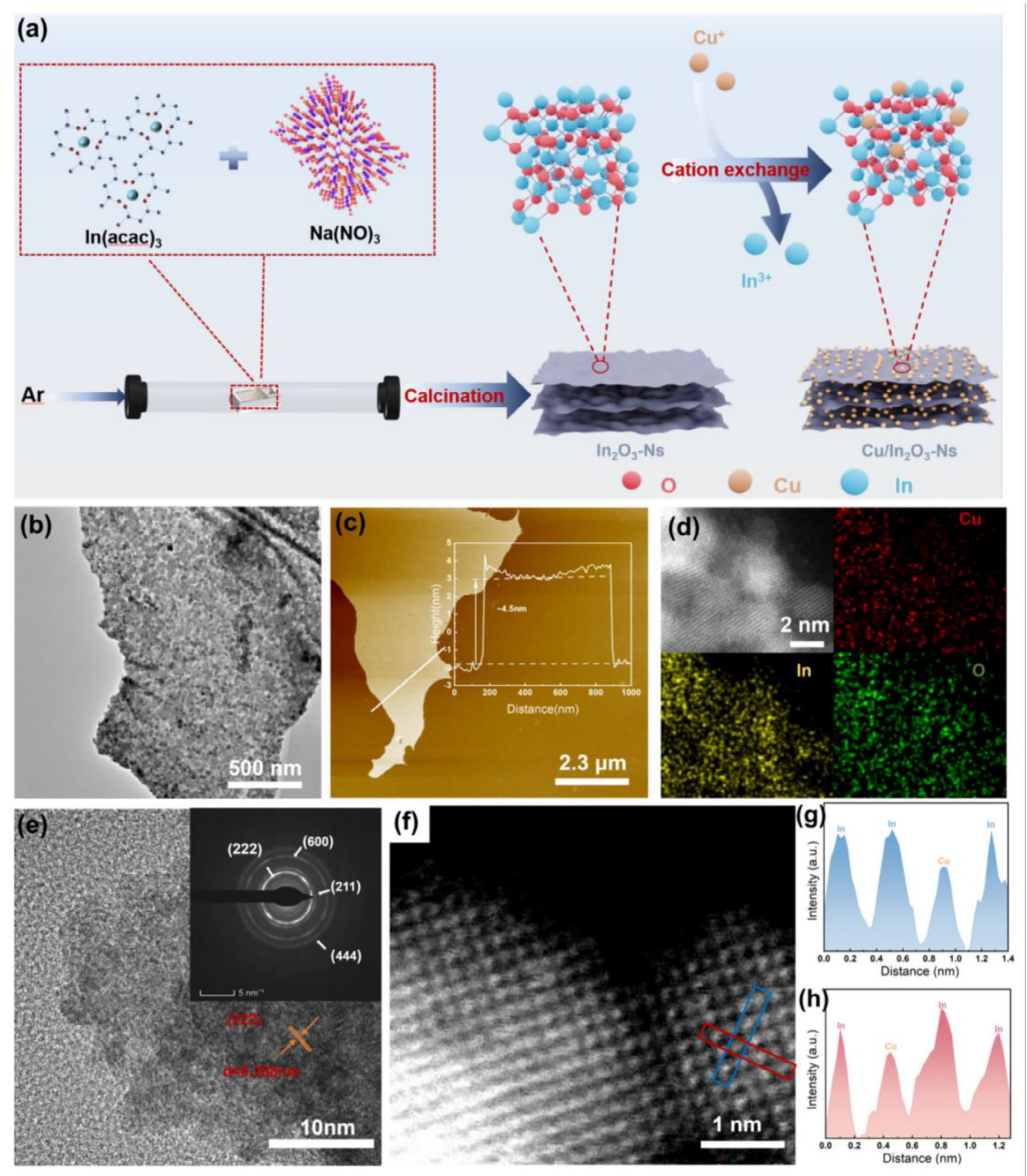
(a) Schematic diagram of the synthesis process for Cu/In_2_O_3_‐Ns; (b) TEM, (c) AFM, (d) HAADF‐STEM and corresponding EDS elemental mapping images of Cu/In_2_O_3_‐Ns; (e) HRTEM image and diffraction ring image of selected regions of Cu/In_2_O_3_‐Ns; (f) AC‐HAADF‐STEM image and corresponding intensity profile in the box of Cu/In_2_O_3_‐Ns; (g) and (h) show the corresponding intensity distributions indicated by the boxes in Figure 1f.

As shown in Figure 1a, a straightforward grinding‐calcination approach was employed to prepare the 2D ultrathin In_2_O_3_‐Ns with abundant oxygen vacancies. Briefly, a stoichiometric mixture of indium (III) acetylacetonate (In(acac)_3_) and sodium nitrate (NaNO_3_) was thoroughly ground into a homogeneous powder, followed by calcination under an Ar atmosphere. During the calcination process, the organic ligand (acetylacetone) of In(acac)_3_ decomposed to release In^3+^, which reacted with sodium oxide generated from the decomposition of NaNO_3_ to form In_2_O_3_. The Ar atmosphere leaded to insufficient oxygen supply, resulting in incomplete oxidation of In^3+^, which caused oxygen vacancies in the generated In_2_O_3_ crystal structure. Subsequently, Cu/In_2_O_3_‐Ns photocatalysts were prepared by cation exchange in ethanol solution using Cu/In equimolar ratios of copper acetate (Cu(CH_3_COO)_2_) and In_2_O_3_‐Ns stirred for 2 h at room temperature. The oxygen vacancies in In_2_O_3_‐Ns provided a site for adsorption and exchange of Cu^2+^ ions, which replaced In^3+^ on the surface of In_2_O_3_ nanosheets to form the Cu/In_2_O_3_‐Ns photocatalyst.

The content of Cu in the synthesized samples (Table S1) was determined to be 10.73 wt.% using inductively coupled plasma emission spectroscopy (ICP‐OES). The X‐ray diffraction (XRD) analysis reveals distinct diffraction peaks corresponding to the cubic phase of In_2_O_3_ (JCPDS No. 06–0416), while no characteristic peaks related to Cu species are observed. Notably, the diffraction patterns of In_2_O_3_‐Ns exhibit significant peak broadening and intensity reduction compared with that of In_2_O_3_‐Bulk (Figure S1a). After the introduction of Cu^2+^ cation, the characteristic diffraction peak at the (222) plane of In_2_O_3_‐Ns exhibits a significant shift toward a lower 2*θ* value (Figure S1b), while In_2_O_3_‐Bulk exhibits negligible peak shifts. This observation indicates that the incorporation of Cu into the In_2_O_3_ lattice generates a high concentration of oxygen vacancies to maintain charge neutrality. These oxygen vacancies significantly disrupt the local lattice periodicity [30]. Meanwhile, the mismatches in Cu─O and In─O bond lengths and electronegativity induce local lattice distortion and strain fields at the doping sites [31]. In synergy with the inherently high specific surface area and abundant structural defects of the ultrathin nanosheets, these factors collectively give rise to lattice expansion [32].

Scanning electron microscopy (SEM, Figure S2a) and Transmission electron microscopy (TEM, Figure S2b,c) images show that Cu/In_2_O_3_‐Bulk has a cubic shape, and the element mapping diagram (Figure S3) shows the uniform distribution of Cu, O, and In in Cu/In_2_O_3_‐Bulk. SEM image (Figure S4) and TEM (Figure 1b) of Cu/In_2_O_3_‐Ns show that Cu/In_2_O_3_‐Ns has a 2D ultrathin lamellar morphology. Furthermore, the atomic force microscopy (AFM) image (Figure 1c) shows a very low Cu/In_2_O_3_‐Ns thickness of ∼4.5 nm, which further clearly confirms the successful preparation of 2D ultrathin Cu/In_2_O_3_‐Ns. The element mapping diagram (Figure 1d) indicates the uniform distribution of Cu, In, and O in the Cu/In_2_O_3_‐Ns photocatalysts. The wide‐area HAADF‐STEM images and EDS elemental maps (Figure S5) confirmed that Cu species were dispersed at the atomic level throughout all tested areas; no distinct Cu clusters or CuO_x_ nanoparticles were observed. In addition, a high‐resolution transmission electron microscope (HRTEM) image and halo diffraction patterns of electron diffraction in selected areas observe the polycrystalline structure of Cu/In_2_O_3_‐Ns (Figure 1e), and the marked diffraction rings coincide with the (222), (211), (444), and (600) crystal planes of In_2_O_3_. The measured lattice spacing is 0.288 nm, which corresponded to the (222) crystal plane of In_2_O_3_ [33]. Aberration‐corrected high‐angle annular dark‐field scanning transmission electron microscopy (AC‐HAADF‐STEM) image (Figure 1f) demonstrates that Cu species are dispersed in the form of isolated atoms on the surface of Cu/In_2_O_3_‐Ns, and no formation of Cu clusters or nanoparticles is observed. Besides, the brightness of the atomic columns depends on the atomic number, and the atomic brightness should be In (49) > Cu (29). The distinct intensity contrast between Cu and In atoms in the corresponding elemental profiles (Figure 1g,h) provides evidence for the substitutional incorporation of Cu atoms into the In_2_O_3_ lattice, and this atomic‐level dispersion creates isolated Cu active sites.

**FIGURE 2:**
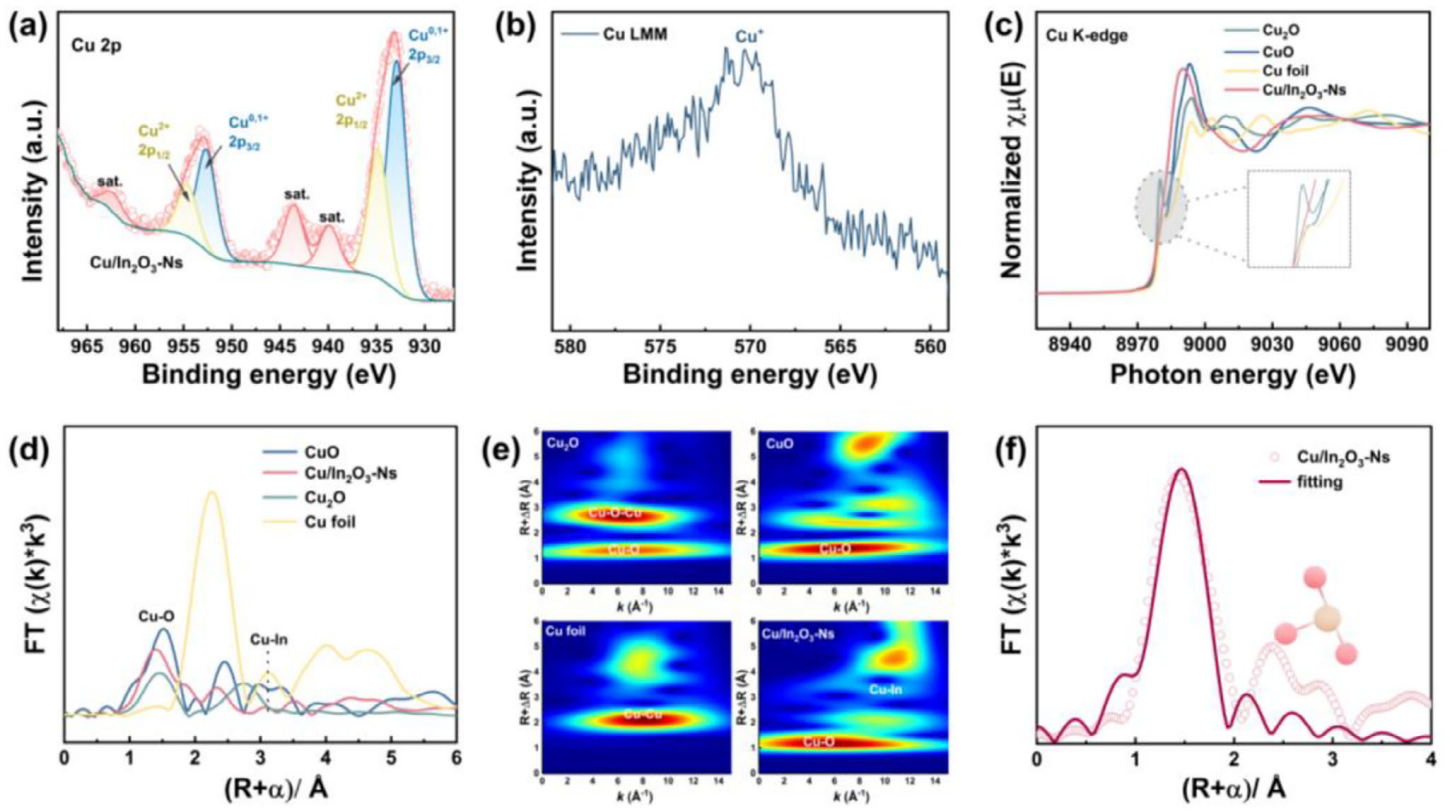
(a) Cu 2p and (b) Cu LMM XPS spectra of Cu/In_2_O_3_‐Ns; (c) Cu K‐edge XANES, (d) Fourier‐transform of the k^3^‐weighted EXAFS and (e) Wavelet transform (WT)‐EXAFS spectra for k^3^‐weighted Cu K‐edge of Cu foil, Cu_2_O, CuO and Cu/In_2_O_3_‐ Ns; (f) fitted FT‐EXAFS spectra of Cu K‐edge for Cu/In_2_O_3_‐Ns and Cu‐O_3_ coordination models.

To elucidate the interaction between Cu single atoms (Cu SAs) and In_2_O_3_ substrate, the chemical states and coordination environments of the dispersed Cu species were systematically investigated by X‐ray photoelectron spectroscopy (XPS), X‐ray absorption near‐edge structure (XANES), and extended X‐ray absorption fine structure (EXAFS) spectra. The Cu 2p XPS spectrum of the Cu/In_2_O_3_‐Ns sample (Figure 2a) exhibits two intense peaks at 933.23 eV (Cu 2p_3/2_) and 953.23 eV (Cu 2p_1/2_), which are attributed to Cu^+^ or Cu^0^, while the two weaker peaks at 935.05 and 955.05 eV correspond to the Cu^2+^ oxidation state. Additionally, the Cu LMM XPS spectrum (Figure 2b) shows a strong peak indicative of Cu^+^ [34, 35]. XPS analysis confirms that the chemical states of Cu species in the Cu/In_2_O_3_‐Ns photocatalyst are between +1 and +2 oxidation states. The normalized Cu K‐edge XANES spectra further confirm the mixed valence states of Cu species. As shown in Figure 2c, the absorption edge of Cu/In_2_O_3_‐Ns is located between those of standard Cu_2_O (Cu^+^) and CuO (Cu^2+^), unambiguously demonstrating the mixed‐valence nature of Cu with an average oxidation state between Cu^+^ and Cu^2+^ [36, 37]. The coordination structure of Cu atoms in the Cu/In_2_O_3_‐Ns photocatalysts was systematically investigated by Fourier‐transform extended X‐ray absorption fine structure (FT‐EXAFS) spectroscopy (Figure 2d and Figure S6). The significant peak at 1.5 Å is caused by direct Cu─O bonding between isolated Cu atoms and lattice oxygen atoms of the In_2_O_3_ support, demonstrating well‐defined Cu–O coordination. The absence of Cu–Cu (∼2.2 Å) scattering characteristics confirms the atomic dispersion of Cu species, which are only coordinated with framework oxygen in the In_2_O_3_ matrix. Notably, the weak peak in Figure 2d at about 3.2 Å is presumed to be a Cu─In bond formed in Cu/In_2_O_3_‐Ns based on previous reports, demonstrating that the isolated Cu atoms form bonds with both lattice oxygen and In atoms of the In_2_O_3_ support [38, 39]. The Wavelet transform WT‐EXAFS analysis (Figure 2e) correlated the FT‐EXAFS peaks with their k‐space data. For the Cu/In_2_O_3_‐Ns photocatalyst, the WT intensity maximum is concentrated at (6.5 Å^−1^,1.5 Å), corresponding to the contribution of Cu–O in the first coordination layer, whereas the Cu‐Cu scattering lobe, which usually occurs at (8.5 Å^−1^, 2.1 Å), is absent. The WT contour plot further confirmed the atomic dispersion of Cu on the Cu/In_2_O_3_‐Ns and the presence of Cu–O–In coordination. The structural parameters of Cu species are quantitatively extracted using EXAFS fitting analysis, and the fitted curves are in good agreement with the experimental spectra [40]. EXAFS fitting analysis (Figure 2f and Table S2) revealed that each isolated Cu atom is coordinated by three O atoms, forming a Cu–O_3_ coordination configuration (inset, Figure 2f). The formation of Cu‐O_3_ units primarily originates from the defective properties of the In_2_O_3_ support, where O_V_ effectively captures and restricts isolated Cu atoms, providing causal support for the formation of Cu‐O_V_‐In disjoint coordination. Therefore, the Cu/In_2_O_3_‐Ns photocatalysts have significant advantages, including an ultrathin 2D structure, abundant oxygen vacancies, Cu SAs sites, In^3+^‐O_V_ sites, and optimized electron distribution to enhance photocatalytic activity.

**FIGURE 3:**
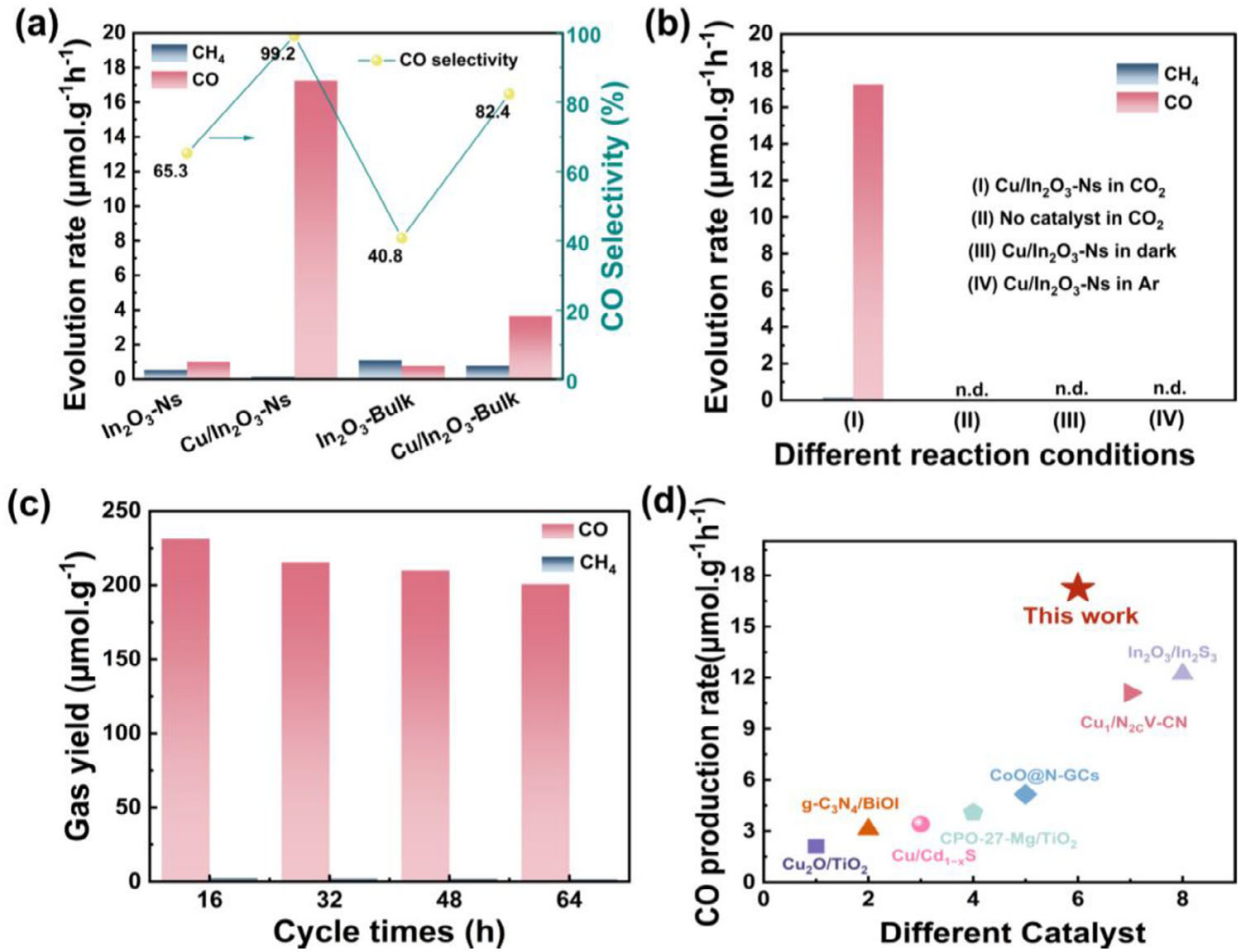
(a) Photocatalytic evolution rates of products for different photocatalysts; (b) Controlled experiments of Cu/In_2_O_3_‐Ns under different conditions; (c) Cyclic testing of Cu/In_2_O_3_‐Ns for photocatalytic CO_2_ reduction reaction; (d) Comparison for the performance of Cu/In_2_O_3_‐Ns with reported photocatalysts for CO production by CO_2_ photoreduction.

The photocatalytic CO_2_ reduction was conducted in a CO_2_‐saturated lactate/water mixed system (1:4 v/v), with the performance of Cu/In_2_O_3_‐Ns and Cu/In_2_O_3_‐Bulk photocatalysts systematically evaluated under full‐spectrum irradiation. As shown in Figure 3a, the Cu/In_2_O_3_‐Ns photocatalysts demonstrate excellent performance in photocatalytic CO_2_ reduction, and the CO production rate reaches 17.243 µmol g^−1^ h^−1^, which is 16.7 and 4.7 times higher than that of In_2_O_3_‐Ns (1.03 µmol g^−1^ h^−1^) and Cu/In_2_O_3_‐Bulk (3.64 µmol g^−1^ h^−1^), respectively, and the CO selectivity is as high as 99.2%. To highlight the role of oxygen vacancies in the photoreduction of CO_2_, a comparison of the photocatalytic performance of Cu/In_2_O_3_‐Ns with O_V_‐poor and O_V_‐rich structures (Figure S7) reveals that the photocatalytic activity of Cu/In_2_O_3_‐Ns with an O_V_‐rich structure is higher, being approximately five times that of the O_V_‐poor structure. Notably, ^1^H NMR spectroscopy analysis (Figure S8) confirms the exclusive formation of gaseous products during photocatalytic CO_2_ reduction over Cu/In_2_O_3_‐Ns, and no by‐products containing liquid carbon are detected. To verify the origin of the products in the photocatalytic CO_2_ reduction process, controlled experiments under different conditions are conducted, including the absence of photocatalysts, light, and CO_2_. The results show that almost no detectable products are observed under all these conditions (Figure 3b), which clearly demonstrates that both CO and CH_4_ originate from the photocatalytic CO_2_ reduction reaction. To further verify the carbon source of the product, a ^13^CO_2_ isotope labelling experiment was carried out. Mass spectrometry analysis revealed a signal peak at m/z = 29 (Figure S9), which corresponds to ^13^CO; this provides strong evidence that the CO product originates from the photoreduction of CO_2_ [41]. Figure S10 shows the apparent quantum yield (AQY) at 370 and 456 nm. In addition, to investigate the stability of the Cu/In_2_O_3_‐Ns photocatalysts in the photocatalytic CO_2_ reduction process, the photocatalyst is subjected to cyclic stability tests. After four cycles totaling 64 h of light irradiation, the CO yield of the Cu/In_2_O_3_‐Ns catalysts does not decay significantly (Figure 3c). XRD and SEM analysis reveal that the crystal structure of Cu/In_2_O_3_‐Ns remains essentially unchanged after the photocatalytic cycling reaction (Figure S11). The AC‐HAADF‐STEM images (Figure S12) taken after the cycle confirm that the copper species remain uniformly dispersed in a single‐atom form on the In_2_O_3_‐Ns surface, with no formation of copper clusters or copper‐based oxide nanoparticles. These results indicate that the Cu/In_2_O_3_‐Ns catalysts exhibit remarkable stability during long‐term photocatalytic operation without detectable structural or compositional alterations. Remarkably, the highly active and selective Cu/In_2_O_3_‐Ns catalysts demonstrate outstanding performance in the photocatalytic conversion of CO_2_ to CO, exhibiting both higher production rates and selectivity compared to most reported photocatalysts (Figure 3d and Table S3) [42, 43, 44, 45, 46, 47, 48].

**FIGURE 4:**
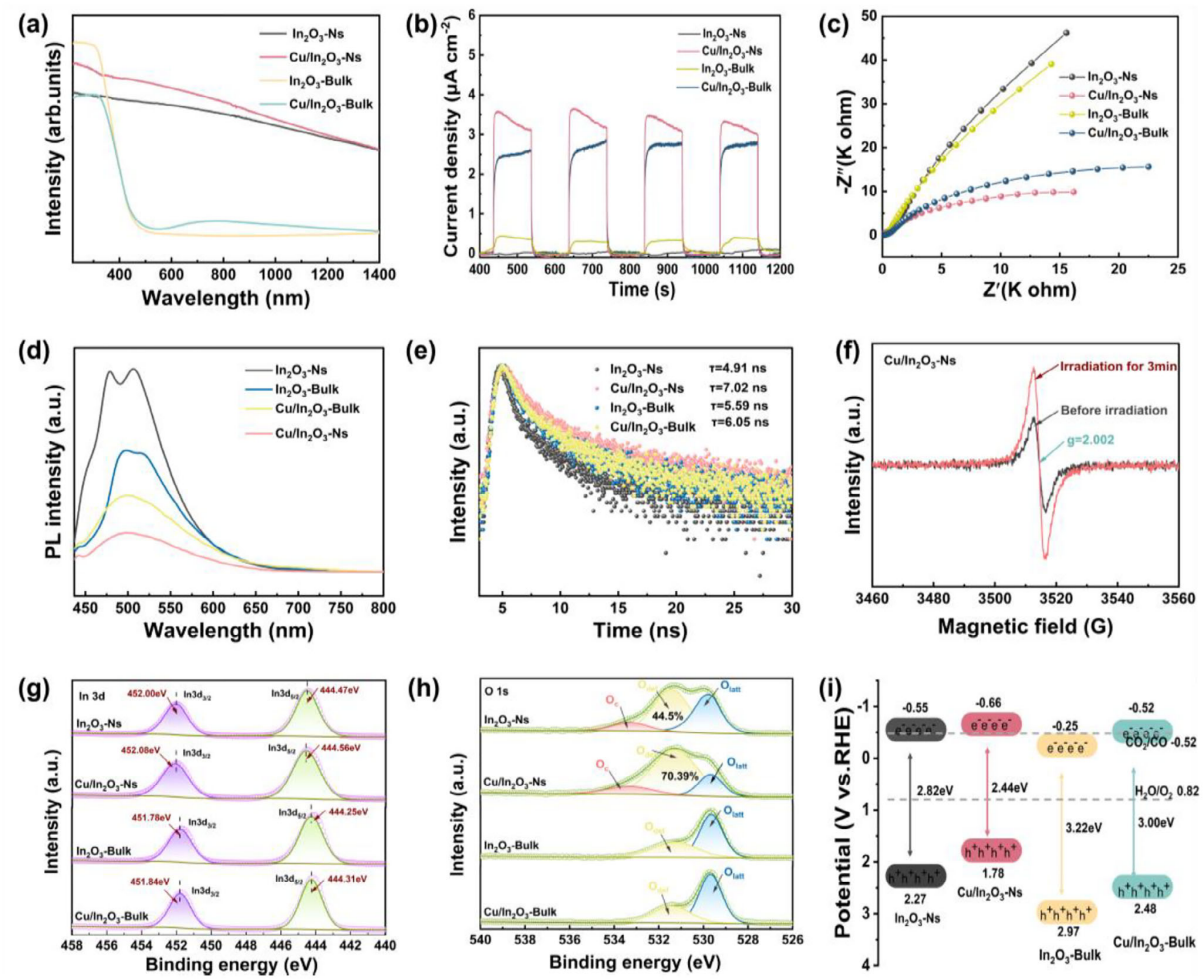
(a) UV–vis–NIR absorption, (b) transient photocurrent response, (c) EIS Nyquist plots, (d) steady‐state PL spectra and (e) time‐resolved PL spectra of In_2_O_3_‐Ns, Cu/In_2_O_3_‐Ns, In_2_O_3_‐Bulk and Cu/In_2_O_3_‐Bulk; (f) EPR spectra of Cu/In_2_O_3_‐Ns; (g) In 3d and (h) O 1s XPS spectra, and (i) energy band diagram of In_2_O_3_‐Ns, Cu/In_2_O_3_‐Ns, In_2_O_3_‐Bulk and Cu/In_2_O_3_‐Bulk.

The photocatalytic efficiency is primarily determined by three key factors: light absorption, separation and transfer of photogenerated carriers, and adsorption/transformation of reactants and intermediates. UV–visible–near–infrared diffuse reflectance spectroscopy (UV–VI–NIR DRS) measurements (Figure 4a) show that the absorption edge of Cu/In_2_O_3_‐Ns exhibits a significantly redshifted in the visible light region, and the light absorption is slightly enhanced compared with the In_2_O_3_‐Ns. The improvement of light absorption performance is attributed to the introduction of a mid‐gap state through Cu incorporation. In the visible‐light region (400–800 nm), the Cu/In_2_O_3_‐Ns sample exhibits significantly enhanced absorption intensity compared to Cu/In_2_O_3_‐Bulk, which could be attributed to its ultrathin 2D structure that provides abundant light‐scattering sites and facilitates photon harvesting, thereby enabling more efficient solar energy utilization.

The isotherms and corresponding pore size distributions of the samples were studied through N_2_ adsorption‐desorption tests (Figure S13 and Table S4). Both In_2_O_3_‐Ns and Cu/In_2_O_3_‐Ns samples show type II adsorption‐desorption isotherms, with predominantly open surfaces and specific surface areas of 56.7376 and 99.2035 m^2^ g^−1^, respectively, and the high specific surfaces are derived from their ultrathin structures and highly exposed surface‐active sites. In contrast, the destruction of the original cubic structure of a typical Cu/In_2_O_3_‐Bulk photocatalyst might lead to the collapse or reorganization of some lattices after cation exchange, reducing the active sites originally exposed on the surface [49]. As a result, its specific surface area decreased from 40.3548 to 22.2546 m^2^ g^−1^ (Figure S14).

Photoelectrochemical measurements (Figure 4b) and electrochemical impedance spectroscopy (EIS) (Figure 4c) indicate that the Cu/In_2_O_3_‐Ns photocatalyst exhibits significantly enhanced charge separation and transfer efficiency. This improvement can be ascribed to the incorporation of Cu single atoms, which optimizes the kinetics of photogenerated carriers. Moreover, the ultrathin 2D confined structure effectively shorts charge diffusion distances, thereby further promoting charge transfer efficiency. To further elucidate the mechanism of charge transfer, photoluminescence (PL) spectroscopy is employed. Steady‐state (Figure 4d) and time‐resolved PL spectra (Figure 4e) analysis indicate that Cu/In_2_O_3_‐Ns exhibits the weakest PL emission intensity and prolonged charge carrier lifetime, indicating that the photogenerated electron‐hole recombination rate is significantly suppressed, which is highly consistent with the photocurrent measurement results.

Electron paramagnetic resonance (EPR) spectroscopy (Figure 4f) reveals a symmetrical signal at g = 2.002 for Cu/In_2_O_3_‐Ns, unambiguously confirming the presence of indium‐oxygen vacancies. This signature further demonstrates that unpaired electrons are captured at these oxygen vacancy sites, which is critical for facilitating charge separation in photocatalytic processes. Upon 3 min irradiation with a 300 W Xe lamp, the Cu/In_2_O_3_‐Ns photocatalyst exhibits a remarkably enhanced oxygen vacancy signal. This phenomenon arises from photoactivated ligand oxygen atoms that cleaved In‐O bonds under illumination, thereby generating additional oxygen vacancies sites. Simultaneously, these vacancies are competitively occupied by gaseous oxygen or its activated species during the photocatalytic process [50, 51]. This mechanism enhances the adsorption and activation of CO_2_, and both Cu sites and O_V_ synergistically promote the photocatalytic reduction of CO_2_ to CO.

Furthermore, XPS tests were employed to investigate the chemical state of indium and oxygen vacancies. The XPS spectra (Figure 4g) confirms that all In species exist in the In^3+^ state. Notably, the incorporation of Cu atoms induces a distinct shift of the In 3d peaks toward higher binding energy compared to pristine In_2_O_3_, indicating a reduced electron density around In^3+^ sites. This electronic redistribution likely enhances the Lewis acidity of surface In^3+^ sites, thereby promoting the adsorption and activation of CO_2_ [52, 53]. It is worth noting that Cu/In_2_O_3_‐Ns exhibits a more obvious binding energy shift (0.08 eV) compared to Cu/In_2_O_3_‐Bulk (0.06 eV), suggesting that uniform Cu doping in the bulk phase likely establishes more stable electron transport pathways, thereby effectively suppressing the recombination of photogenerated carriers. The O 1s XPS spectrum (Figure 4h) exhibits three deconvoluted peaks at approximately 533.33, 531.42, and 529.73 eV, corresponding to surface hydroxyl groups (‐OH, O_C_), defect‑related oxygen species (O_def_), and lattice oxygen (O_latt_), respectively. Notably, the In_2_O_3_‐Ns shows a higher proportion of chemisorbed oxygen species compared to In_2_O_3_‐Bulk, which is conducive to the activation and adsorption of CO_2_ due to its enhanced surface reactivity [54]. Quantitative analysis of the peak area ratio (O_def_/[O_C_+O_def_+O_latt_]) indicates that 70.4% of oxygen defects in Cu/In_2_O_3_‐Ns exists as O_V_, which is significantly increased compared with 44.5% in pristine In_2_O_3_. This striking enhancement directly proves the Cu induces the generation of oxygen vacancies. The increased O_def_ fraction observed in XPS, combined with the strong paramagnetic signal in EPR, reliably confirms the presence of abundant oxygen vacancies in the catalyst. This is consistent with the EPR results. Furthermore, based on Tauc plots (Figure S15), Mott‐Schottky measurements (Figure S16), and the empirical relationship defining the bandgap (E_g_), valence band (E_VB_), and conduction band (E_CB_) positions (E_g_ = E_VB_ − E_CB_), the derived energy band structures of the four samples were derived as shown in Figure 4i. Notably, Cu/In_2_O_3_‐Ns exhibits the most favorable conduction and valence band alignment, which thermodynamically facilitated both CO_2_ reduction and H_2_O oxidation half‐reactions.

**FIGURE 5:**
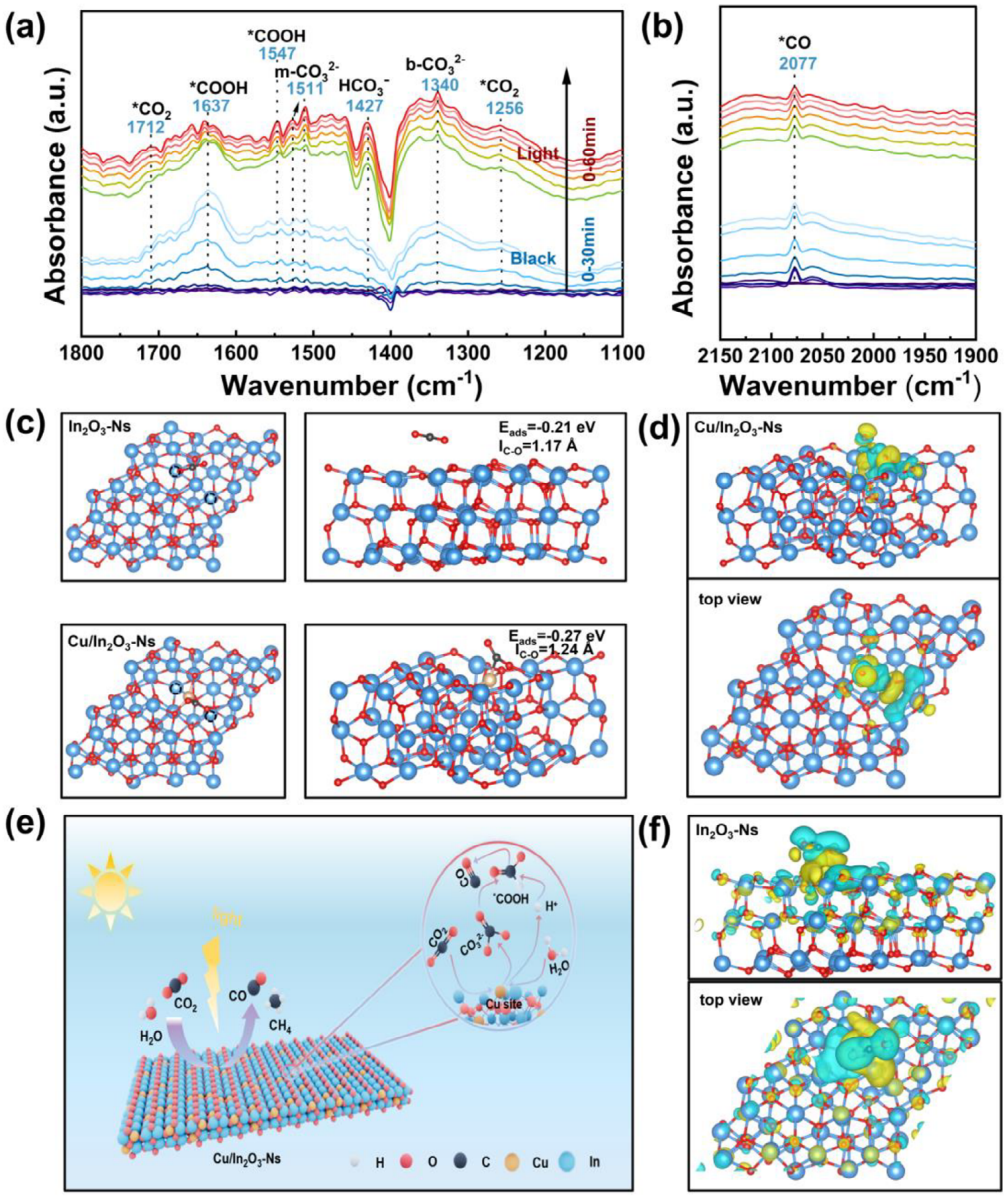
(a,b) In situ drift spectra for different irradiation times during CO_2_ photoreduction on Cu/In_2_O_3_‐Ns; (c) DFT calculated models and comparative adsorption configurations and energies of CO_2_: In_2_O_3_‐Ns (111) and Cu/In_2_O_3_‐Ns (111) surfaces (black circles: oxygen vacancies, blue: In, red: O, yellow: Cu, black: C); (d) charge density difference and corresponding charge transfer for CO_2_ adsorbed Cu/In_2_O_3_‐Ns. The yellow and blue regions represent the accumulation and depletion of electrons, respectively; (e) photoreduction mechanism diagram of Cu/In_2_O_3_‐Ns; (f) charge density difference and corresponding charge transfer for CO_2_ adsorbed In_2_O_3_‐Ns.

To clarify the reaction pathway of photocatalytic conversion of CO_2_‐to‐CO, in situ diffuse reflectance infrared Fourier transform spectroscopy (DRIFTS) was employed to track the evolution of reactive intermediates during the CO_2_ photoreduction process. In the photocatalytic system employing the Cu/In_2_O_3_‐Ns photocatalyst, the reaction was first allowed to be stirred continuously for 30 min in the dark to achieve adsorption equilibrium. Upon subsequent irradiation, a gradual increase in infrared signals is observed with prolonged illumination time (Figure 5a,b), indicating the light‐driven activation process. The peak at 1511 cm^−1^ is associated with monodentate carbonate (m‐CO_3_^2−^) species, while the peak at 1340 cm^−1^ corresponds to bidentate carbonate (b‐CO_3_^2−^) adsorption [54, 55]. The characteristic ^*^CO_2_ vibration bands observed at 1256 and 1712 cm^−1^ can be ascribed to the photo‐oxidation process of CO_2_. In addition, bicarbonate (HCO_3_^−^) production is observed at 1427 cm^−1^ [56]. Notably, hydroxyl (‐OH) groups on the Cu/In_2_O_3_‐Ns surface can interact with adsorbed CO_2_ to form HCO_3_^−^ species, synergistically promoting CO_2_ adsorption and activation with In^3+^‐O_V_ vacancy sites. In situ DRIFTS analysis confirmed the formation of CO_3_^2−^ and HCO_3_^−^ intermediates, further confirming the enhanced CO_2_ adsorption capacity of the Cu/In_2_O_3_‐Ns photocatalyst. Under light irradiation, the CO_2_ photoreduction reaction accelerated the entire process of CO_2_ adsorption, intermediate formation, and CO/CH_4_ generation/desorption. It is worth noting that the characteristic peaks at 1547 and 1637 cm^−1^ were assigned to the stretching vibrations of the ^*^COOH group, which is widely recognized as a key intermediate in the photocatalytic reduction of CO_2_ to CO or CH_4_ [53, 57]. In contrast, the spectrum of In_2_O_3_‐Ns (Figure S17) showed almost no relevant characteristic peaks, further confirming that the Cu‐O_V_‐In sites promote the formation of ^*^COOH rather than merely enhancing CO_2_ adsorption. It is worth noting that the H─O─H bending vibration of adsorbed water remains stable in the dark state, which cannot explain the light‐dependent increase; carbonate species reach adsorption saturation during the dark adsorption phase, indicating a fundamental difference in their kinetic behavior; simultaneously, the spectra did not detect characteristic fingerprint peaks of formate (C─H stretching vibrations at 1380, 1580 cm^−1^), ruling out the possibility of a formate reaction pathway [58, 59, 60]. The intensity of the ^*^CO adsorption peak gradually increased with the extension of irradiation time, accompanied by a gradual rise in CO concentration. In addition, the peak at 2077 cm^−1^ is attributed to the CO^*^ adsorption band, and the intensity of the CO^*^ adsorption peak with gaseous CO is gradually enhanced with the increase of irradiation time, indicating that a large number of CO intermediates can be generated by constructing the Cu‐O_V_‐In interface, which leads to the volatilization of more CO gas [61].

Proton supply plays a critical role in governing the reaction kinetics of photocatalytic CO_2_ reduction, which is strongly dependent on the water dissociation capacity of the catalyst surface. In‐situ DRIFTS was employed to track the dynamic evolution of O─H stretching vibrations in the 3000–3700 cm^−1^ region on Cu/In_2_O_3_‐Ns during the reaction (Figure S18). The progressively intensified absorption in this range originates from both surface hydroxyl species and adsorbed water molecules: two sharp peaks at 3627 and 3597 cm^−1^ are assigned to free terminal In‐OH groups on coordinatively unsaturated sites, which feature high reactivity due to the absence of intermolecular hydrogen bonds, while the broad absorption band at 3200–3550 cm^−1^ corresponds to hydrogen‐bonded hydroxyl networks and adsorbed water with diverse configurations [62, 63]. As the dissociation activation energy of O–H species is positively correlated with hydrogen bond strength, the abundant weakly hydrogen‐bonded O─H^δ+^ active species on Cu/In_2_O_3_‐Ns give rise to a lowered water dissociation barrier, which sustains sufficient proton supply for proton‐coupled electron transfer steps such as ^*^COOH formation [64, 65]. This characteristic explains the catalyst's outstanding carbon dioxide reduction and water oxidation performance and can be attributed to the synergistic effect between the enriched surface oxygen vacancies which serve as active sites for water adsorption and dissociation and the optimized efficiency of photogenerated charge separation.

To gain a deeper understanding of the mechanism of the CO_2_ photoreduction reaction of Cu/In_2_O_3_‐Ns, density functional theory (DFT) calculations were performed. First‐principles calculations were performed to clarify the electronic structure of Cu/In_2_O_3_‐Ns. To investigate the synergistic effects of the Cu‐O_V_‐In interface structure in the photocatalytic reduction of CO_2_ to CO, we optimized the adsorption configurations of CO_2_ molecules on the surfaces of In_2_O_3_‐Ns and Cu/In_2_O_3_‐Ns (Figure 5c). The oxygen atom of the CO_2_ molecule interacts with the low‐valence indium atom exposed in the oxygen vacancy, while the carbon atom interacts with the Cu single atom. This “dual‐site anchoring” mode significantly enhances the adsorption capacity of CO_2_ and elongates the C═O bond, thereby facilitating its reduction and activation. The adsorption energy of CO_2_ molecules on the Cu‐O_V_‐In sites (ΔE_ads_ =−0.27 eV) is more negative than that on the In‐O_V_‐In sites (ΔE_ads_ =−0.21 eV), indicating that the Cu/In_2_O_3_‐Ns catalysts have high chemisorption reactivity. It is noteworthy that the C═O bond lengths at the In‐O_V_‐In and Cu‐O_V_‐In sites are 1.17 and 1.24 Å, respectively, indicating that the CO_2_ molecule is more active at the Cu‐O_V_‐In site and is more prone to breakage.

To elucidate the electron distribution at the Cu‐O_V_‐In site after CO_2_ adsorption, charge density differences were calculated. At the In‐O_V_‐In site following CO_2_ adsorption, a distinct electron accumulation at the O_V_ site is clearly observable (Figure 5f). However, no significant charge accumulation is observed at the O_V_ site of the Cu‐O_V_‐In site, demonstrating that more electrons are transferred to the CO_2_ molecule via the Cu‐O_V_‐In site (Figure 5d). This provides theoretical support for the adsorption and activation of CO_2_ molecules at the In^3+^‐O_V_, while the Cu single atom acts as an electron relay station to receive photo‐generated electrons and supply electrons to the activated CO_2_, forming the ^*^COOH intermediate. The two are tightly coupled at the interface, synergistically constructing a highly efficient and selective catalytic microenvironment that optimizes the transport processes of electrons and protons, respectively. This electronic configuration enhanced the charge transfer capability of Cu/In_2_O_3_‐Ns, thereby significantly improving the photocatalytic kinetics.

These results demonstrate that Cu/In_2_O_3_‐Ns preferentially promotes the photoreduction of CO_2_, highlighting its selectivity toward solar‐driven CO_2_ conversion. This shows that the introduction of Cu single atoms effectively reduces the kinetic barrier for CO_2_ reduction to ^*^COOH, highlighting the critical role of Cu in regulating the reaction pathway. This might be attributed to the strong interaction between the Cu‐O_V_‐In, which promotes the stabilization of the ^*^COOH intermediate, reduces the activation energy of CO_2_, and ultimately increases the yield of CO. Moreover, the observed high CO activity and selectivity might be attributed to the rapid dissociation of ^*^CO species on the Cu/In_2_O_3_‐Ns surface, facilitating the effective desorption of CO. In situ DRIFTS studies reveal that the photocatalytic CO_2_ reduction pathway over Cu/In_2_O_3_‐Ns proceeds via the following sequence: CO_2_ → CO_3_^2−^ → ^*^COOH → ^*^CO → CO. The proposed mechanistic evolution is shown in Figure 5e, demonstrating the key intermediates stabilized by the unique electronic structure of Cu/In_2_O_3_‐Ns during the CO_2_ photoreduction process.

## 3 Conclusion

In summary, we have successfully synthesized ultrathin two‑dimensional Cu/In_2_O_3_‑Ns via a room‑temperature cation‑exchange strategy. The crux of the enhanced photocatalytic CO_2_ reduction lies in the formation of a Cu‐O_V_‐In interfacial architecture, which originates from the strong electronic interaction between Cu single atoms and the In_2_O_3_ host. This interface synergistically couples the benefits of the ultrathin 2D confinement, abundant oxygen vacancies, and atomically dispersed Cu sites: it promotes CO_2_ enrichment and activation, stabilizes the key COOH intermediate, and accelerates charge separation and electron transfer. As a result, the Cu/In_2_O_3_‑Ns catalyst delivers remarkably high CO production activity and selectivity. Our work not only demonstrates the power of interfacial engineering in designing single‑atom photocatalysts, but also offers a fundamental perspective on how the Cu‐O_V_‐In motif governs both activity and selectivity in CO_2_ photoreduction.

## Author Contributions

**Ying Bian**: data curation. **Jiali Ren**: validation. **Porun Liu**: writing – review and editing. **Cheng‐Hao Chuang**: writing – review and editing. **Xuan Xiang**: investigation. **Xiaoli Zhang**: writing – review and editing. **Xiaoyang Lu**: resources. **Yueying Yang**: writing – original draft. **Xinyu Wang**: visualization. **Yanjun Xue**: writing – review and editing. **Jian Tian**: writing – review and editing .

## Conflicts of Interest

The authors declare no conflicts of interest.

## Supporting information




**Supporting File**: advs77464‐sup‐0001‐SuppMat.docx.

## Data Availability

The data that support the findings of this study are available from the corresponding author upon reasonable request.
