# Supplementary material for: Ultrathin 2D In2O3 Nanosheets Confining Cu Single Atoms Synergized With Oxygen Vacancies for High Selectivity of Photocatalytic Reduction of CO2 to CO
Source: Adv Sci (Weinh). 2026 Aug 30:e77464. Online ahead of print. doi: 10.1002/advs.77464 (PMC13526707; doi:10.1002/advs.77464)
Supplement: Supplementary file 1 [file ADVS-9999-e77464-s001.docx]

Supporting Information

Ultrathin 2D In_2_O_3_ Nanosheets Confining Cu Single Atoms Synergized with Oxygen Vacancies for High Selectivity of Photocatalytic Reduction of CO_2_ to CO

*Yueying Yang^a^, Xuan Xiang^a^, Jiali Ren^a^, Ying Bian^b^, Xinyu Wang^a^, Xiaoyang Lu^a^, Yanjun Xue^*, a^, Porun Liu^*, c^, Cheng-Hao Chuang^*, d^, Xiaoli Zhang^*^*^,^ *^e^, Jian Tian^*^*^,^ *^a^*

*^a^ State Key Laboratory of Disaster Prevention and Ecology Protection in Open-pit Coal Mines, Shandong Key Laboratory of Special Epoxy Resin, School of Materials Science and Engineering, Shandong University of Science and Technology, Qingdao 266590, China*

*^b^ Dongying Vocational College, Dongying 257091, China*

*^c^ School of Environment and Science, Gold Coast Campus, Griffith University, Gold Coast, Queensland 4222, Australia*

*^d^ Department of Physics, Tamkang University, Tamsui 251, New Taipei City, Taiwan*

*e Australian Research Council Centre of Excellence for Green Electrochemical Transformation of Carbon Dioxide, Applied Chemistry and Environmental Science, School of Science, RMIT University, Melbourne VIC 3001, Australia*

** Corresponding authors: xueyuan196311@163.com (Y. Xue); p.liu@griffith.edu.au (P. Liu); chasehao@gmail.com (C. Chuang); xiaoli.zhang2@rmit.edu.au (X. Zhang); jiantian@sdust.edu.cn (J. Tian)*

**Experimental Procedures**

**Chemicals**

All chemicals were purchased from the market without further purification, including indium nitrate hydrate (In(NO_3_)_3_-xH_2_O, 99.99% metal-based), copper acetate ((CH_3_COO)_2_Cu·H_2_O), sodium nitrate (NaNO_3_, analytically pure), indium acetylacetonate (C_15_H_21_InO_6_), anhydrous ethanol (CH_3_CH_2_OH, analytically pure), urea (CO(NH_2_)_2_, analytically pure), deionized water, DL-lactic acid (C_3_H_6_O_3_, 99%).

**Synthesis of In_2_O_3_-Ns**

0.15 g of indium acetylacetonate and 10 g of sodium nitrate were weighed into an agate mortar and ground thoroughly for one hour to obtain a mixed powder. Then, the ground mixture was placed into an alumina porcelain boat in a tube furnace passed through argon atmosphere and heated to 270 °C at 2 °C/min and kept for one hour. After cooling to room temperature, the mixture was washed several times alternately with water and ethanol centrifugation and finally dried under vacuum at 60 °C overnight. Under otherwise identical conditions, substituting argon with air yielded In_2_O_3_-Ns without oxygen vacancies.

**Synthesis of In_2_O_3_-Bulk**

In the typical In_2_O_3_ synthesis, it was prepared by hydrothermal method. 6 mmol In(NO_3_)_3_-xH_2_O was added to 40 mL of aqueous solution. After stirring for 10 min at room temperature, 24 mmol of urea was added and stirred for 2 h. The mixture solution was then transferred to a 50 mL Teflon-lined autoclave and kept at 140 °C for 16 hours. The resulting solid product was washed thoroughly with water and ethanol to remove residues and then dried under vacuum at 60 °C overnight.

**Synthesis of Cu/In_2_O_3_-Ns and Cu/In_2_O_3_-Bulk**

At ambient temperature, copper acetate was dissolved in an appropriate amount of ethanol solution and added drop by drop to an ethanol solution of In_2_O_3_-Ns or In_2_O_3_-Bulk, and then stirred for 2 h. The copper-indium molar ratio of copper acetate and copper-indium of the substrate was 1:1. The resulting mixed solution was then centrifuged, washed several times alternately with water and ethanol, and finally dried in a vacuum drying oven at 60 °C overnight to obtain Cu/In_2_O_3_-Ns and Cu/In_2_O_3_-Bulk catalysts.

**Characterization**

The XRD patterns of the synthesized products were measured on a Rigaku Utima IV diffractometer (Rigaku, Japan). X-ray diffraction (XRD) spectra were measured and collected using an X’Pert PRO (PANalytical) powder diffractometer using a Cu K^α^ radiation source with a wavelength λ of 0.15418 nm, a test voltage of 40 kV, and a current of 40 mA. The in situ diffuse reflectance infrared transform spectroscopy (DRIFT) measurements were performed on an FTIR spectrometer (Thermo, Nicolet iS 50) with an MCT detector. The spectra were displayed in transmission units and acquired with a resolution of 4 cm^-1^, using 64 scans. The dome of the reaction cell had two ZnSe windows allowing IR transmission and a third window allowing transmission of irradiation introduced through a liquid light guide that connects to the same IR-light lamp. The nanostructure and surface characteristics of the samples were tested by high-resolution scanning electron microscopy (FESEM, Apreo S HiVac, USA). High-resolution transmission electron microscopy (HRTEM) was recorded on a Thermo Fisher Talos F200X (USA) transmission electron microscope. Atomic-scale high-angle annular dark-field scanning transmission electron microscopy (HAADF-STEM) and energy dispersive x-ray spectroscopy (EDS) elemental maps were obtained using a Thermo Scientific Themis Z Transmission Electron Microscope (USA) operating at 200 kV and equipped with a double spherical aberration (Cs) corrector. Subsequent correlation analysis was performed using Digital Micrograph software. X-ray photoelectron spectroscopy (XPS) was performed using a Thermo Fisher ESCALAB 250 Xi photoelectron spectrometer equipped with an Al K Alpha excitation source. The peak position was corrected according to C 1s at 284.8 eV. AFM images of Cu/In_2_O_3_-Ns were captured using a Bruker Multi Mode 8 (Bruker, USA) equipped with a Nano Scope V controller. ICP-OES analysis was performed on an Agilent ICP OES 725 ES. The UV-Vis absorption spectrum was carried out by the UV-3101 UV-Vis-NIR spectrophotometer. Electron paramagnetic resonance (EPR) spectra were measured with an EPR spectrometer (BRUKE A300) at 300K and 9.65GHz. Specific surface area and pore size analyses were performed using an ASAP2460 Physical Adsorption Meter (McMorrettick, USA) to analyze the specific surface area and pore size distribution of the materials. The ^1^H nuclear magnetic resonance (NMR) spectrum was measured with a Bruker 400 MHZ nuclear magnetic resonance spectrometer, and the chemical shift in the spectrum was calibrated with deuterated dimethyl sulfoxide. Stable photoluminescence (PL) and time-resolved photoluminescence (TRPL) spectra were measured at an excitation wavelength of 500 nm using an Edinburgh FLS-1000 spectrophotometer. Sotope ratio mass spectrometry (IRMS) measurements were performed on a Thermo Finnigan Delta V Advantage mass spectrometer coupled with a Thermo Finnigan Mat 253 device.

**Computational method and models**

All density functional theory (DFT) simulations were implemented with the Vienna Ab-initio Simulation Package (VASP) [1]. Core-electron interactions were modeled through the projector-augmented wave (PAW) formalism [2], while the generalized gradient approximation (GGA) framework was adopted to approximate exchange-correlation effects using the Perdew-Burke-Ernzerhof (PBE) functional [3]. The plane-wave basis set was truncated at a kinetic energy cutoff of 450 eV, with geometric relaxation continuing until total energy fluctuations fell below 1 × 10⁻^5^ eV and atomic forces reduced to 0.02 eV/Å. A 15 Å vacuum buffer was imposed perpendicular to the material plane to suppress periodic boundary artifacts. Reciprocal space sampling was conducted with a Γ-centered 3 × 3 × 1 k-point grid. The DFT-D3 method was also employed to describe the van der Waals interactions [4].

**Photocatalytic CO_2_ conversion measurements**

Photocatalytic measurements were performed in a photocatalytic activity evaluation system (CEL-PAEM-D8, Beijing Zhongjiao Jinyuan Science and Technology Co). In typical measurements, 10 mg of catalyst was dispersed in a mixed solution containing 40 mL of water and 10 mL of DL-lactic acid. The suspension was loaded into a 150 mL photoreaction vessel containing magnets, and the reactor was evacuated by vacuuming to exclude air interference, and then a saturated atmosphere was created by pumping 0.7-0.8 kpa of high-purity CO_2_ into the reacting photocatalytic glass system. The photoreactor was then placed in a cooling unit maintained at 25 °C by a recirculating cooling water system, stirred, and irradiated with a xenon lamp (Beijing Zhongjiao Jinyuan Science and Technology Co, Ltd, CEL-HXF300) with a power of 300 W for 16 hours. The gas products were analyzed and quantified by a gas chromatograph (GC7920-TF2A).

**Apparent Quantum Efficiency (AQY)**

The apparent quantum yield (AQY) measurements were carried out under monochromatic light at 370 nm and 456 nm, and the corresponding AQY values were calculated from the‑produced CO amount according to the classic photon‑consumption equation. The calculation formula is as follows:

AQY = $\frac{2\times n_{CO}\times N_{A}}{N}$×100%	(1)

N = $\frac{EA_{R}t\lambda}{hc}$	(2)

**Calculate the selectivity of CO**

The selectivity of carbon dioxide reduction to carbon monoxide is calculated by the following equation:

Product selectivity =$\frac{Product yield}{Total product yield of CO and {CH}_{4}}$×100%	(3)

**Photoelectrochemical (PEC) measurements**

The PEC characteristics were measured on a standard three-electrode system with FTO as the working electrode, Pt wire as the counter electrode and Ag/AgCl as the reference electrode in 0.5 M Na_2_SO_4_ aqueous solution. Amperometric current-time (I-t) curves were recorded by an electrochemical workstation (CHI660E, Chenhua, Shanghai, China) under the on/off cycle of a 300 W xenon lamp (Shanghai Hefan Instrument Co., Ltd., HF-GHX-XE-300). EIS Nyquist plots with an amplitude of 5 mV and frequencies ranging from 0.01 Hz to 100 kHz were acquired under open-circuit conditions. The Mott-Schottky plots were measured in the dark at an AC frequency of 1.0 kHz.

**Table S1.** Results of inductively coupled plasma mass spectrometry analysis.

| **Catalyst** | **Cu Percentage (wt.%)** |
| --- | --- |
| Cu/In_2_O_3_-Ns | 10.73 |

**Figure S1.** XRD patterns of In_2_O_3_-Ns, Cu/In_2_O_3_-Ns, In_2_O_3_-Bulk and Cu/In_2_O_3_-Bulk. (a) complete pattern (b) partially enlarged pattern.

**Figure S2.** (a) SEM, (b) TEM, and (c) HRTEM images of Cu/In_2_O_3_-Bulk.

**Figure S3.** TEM-EDS images of Cu/In_2_O_3_-Bulk.

**Figure S4.** SEM image of Cu/In_2_O_3_-Ns.

**Figure S5.** HAADF-STEM image and EDS elemental map of Cu/In_2_O_3_-Ns at 10 nm.

**Figure S6.** Cu K side-extended XAFS spectra and fitting results for the Cu/In_2_O_3_-Ns sample in K-space (FT magnitude and imaginary component). Data are k3-weighted and not phase-corrected.

**Table S2.** Structural parameters of the Cu K-edge EXAFS spectrum (S0^2^ = 0.90) for the Cu/In_2_O_3_-Ns catalyst.

| **Path** | **CN^a^** | **R(Å)^b^** | **σ^2^×10^-3^(Å^2^)^c^** | **ΔE(eV)^d^** | **R factor** |
| --- | --- | --- | --- | --- | --- |
| Cu-O | 3.4±0.4 | 1.92±0.03 | 6.0# | -0.89±3.59 | 0.018 |

^a^CN: coordination number; ^b^R: bond length; ^c^σ^2^: Debye-Waller factors; ^d^ΔE: the inner potential correction. R factor: goodness of fit. To gain best fitting result, the Debye-Waller factors were set to 0.018 for Cu-O path.

**Figure S7.** Photocatalytic production rates of Cu/In_2_O_3_-Ns catalysts with oxygen-rich and oxygen-poor sites.

**Figure S8.** ^1^H-NMR hydrogen spectrum of Cu/In_2_O_3_-Ns.

**Figure S9.** Mass spectrometry spectrum of the photocatalytic reduction of ^13^CO_2_ to ^13^CO (m/z = 29) on Cu/In_2_O_3_-Ns.

**Figure S10.** Apparent Quantum Efficiency of Cu/In_2_O_3_-Ns at Different Wavelengths.

**Figure S11.** (a) XRD patterns of Cu/In_2_O_3_-Ns catalyst before (fresh) and after (used) 4 cycles; (b) SEM image of used Cu/In_2_O_3_-Ns.

**Figure S12.** AC-HAADF-STEM images of Cu/In_2_O_3_-Ns after cycling.

**Table S3.** The catalytic activity of Cu/In_2_O_3_-Ns is compared with the catalytic activity and selectivity of recently reported catalysts for the photoreduction of CO_2_ to CO in simulated sunlight.

| **Photocatalyst** | **Sacrificial agent** | **Light source** | **Products (μmol g^-1^ h^-1^)** | **Selectivity (%)** | **Reference** |
| --- | --- | --- | --- | --- | --- |
| Cu/In_2_O_3_-Ns | Lactic acid | 300 W Xe lamp (AM 1.5 filte) | 17.24 | 99.2 | This work |
| Cu/Cd_1−x_S | H_2_O | 300 W Xe lamp (λ＞420 nm) | 3.4 | 98 | [5] |
| CPO-27-Mg/TiO_2_ | H_2_O | 300 W Xe lamp | 4.09 | 32.1 | [6] |
| CoO@N-GCs | H_2_O | 350 W Xe lamp (λ＞420 nm) | 5.16 | 33.9 | [7] |
| Cu_1_/N_2C_V-CN | H_2_O | 300 W Xe lamp (λ＞420 nm) | 11.12 | 98.5 | [8] |
| Ni-tp-COF | H_2_O | 300 W Xe lamp (λ＞420 nm) | 15 | 99.1 | [9] |
| Cu-MOF-Fcdc-20% | H_2_O | 300 W Xe lamp (λ＞420 nm) | 8.61 | 97.07 | [10] |
| BiOBr_0.5_Cl_0.5_/WO_3_ | H_2_O | 300 W Xe lamp | 16.68 | \ | [11] |
| FeWO_4_/NiAl-LDH | H_2_O | 300 W Xe lamp (λ＞420 nm) | 5 | 93 | [12] |
| mpg-C_3_N_4_ | TEOA | 300 W Xe lamp (AM 1.5 filte) | 8 | 97 | [13] |

**Figure S13.** N_2_ adsorption-desorption curves of (a) In_2_O_3_-Ns and (b) Cu/In_2_O_3_-Ns. Inset shows the pore size distribution.

**Table S4.** BET specific surface area, pore volume and average pore size of different catalysts determined by N_2_ adsorption-desorption measurements.

| **Sample** | **BET surface area (m^2^/g)** | **Pore Volume (cm^3^/g)** | **Average pore size (nm)** |
| --- | --- | --- | --- |
| In_2_O_3_-Ns | 56.73 | 0.14 | 13.01 |
| Cu/In_2_O_3_-Ns | 99.20 | 0.24 | 15.25 |
| In_2_O_3_-Bulk | 40.35 | 0.05 | 8.74 |
| Cu/In_2_O_3_-Bulk | 22.25 | 0.03 | 10.26 |

**Figure S14.** N_2_ adsorption-desorption curves of (a) In_2_O_3_-Bulk and (b) Cu/In_2_O_3_-Bulk. Inset shows the pore size distribution.

**Figure S15.** Tuac plots for different catalysts.

**Figure S16.** Mott-Schottky plots of different catalysts measured at 500, 1000 and 2000 Hz.

**Figure S17.** *In-situ* drift spectra for different irradiation times during CO_2_ photoreduction on In_2_O_3_-Ns.

**Figure S18.** In situ DRIFTS spectra of H_2_O adsorption and activation on Cu/In_2_O_3_-Ns in UV-Vis light and darkness over time.

**References**

1. J. F. G. Kresse, "Efficiency of Ab-Initio Total Energy Calculations for Metals and Semiconductors Using a Plane-Wave Basis Set," Computational Materials Science 6, no. 1 (1996): 15, <https://doi.org/10.1016/0927-0256(96)00008-0>.

2. G. Kresse, D. P. Joubert, "From Ultrasoft Pseudopotentials to the Projector Augmented-Wave Method," Physical Review B 59, no. 3 (1999): 1758, [https://doi.org/10.1103/PhysRevB.59.1758](https://link.wtturl.cn/?target=https%3A%2F%2Fdoi.org%2F10.1103%2FPhysRevB.59.1758&scene=im&aid=582478&lang=zh).

3. J. Perdew, K. Burke, M. Ernzerhof, "Generalized Gradient Approximation Made Simple," Physical Review Letters 77, no. 18 (1996): 3865, [https://doi.org/10.1103/PhysRevLett.77.3865](https://link.wtturl.cn/?target=https%3A%2F%2Fdoi.org%2F10.1103%2FPhysRevLett.77.3865&scene=im&aid=582478&lang=zh).

4. R. H. Dion M, Schröder E, Langreth DC, Lundqvist BI, "Van der Waals Density Functional for General Geometries," Physical Review Letters 92, no. 24 (2004): 246401, <https://doi.org/10.1103/PhysRevLett.92.246401>.

5. H. Cao, X. Zhu, J. Xue, R. Wang, J. Shang, P. Ma, C. Liu, J. Bao, "Defect-Mediated Cu–S Pair Active Sites Modulating Proton Supply to Facilitate Overall CO_2_ Photoreduction with H_2_O," ACS Catalysis 14, no. 13 (2024): 9734, <https://doi.org/10.1021/acscatal.4c02857>.

6. M. Wang, D. Wang, Z. Li, "Self-Assembly of CPO-27-Mg/TiO_2_ Nanocomposite with Enhanced Performance for Photocatalytic CO_2_ Reduction," Applied Catalysis B: Environmental 183, (2016): 47, <https://doi.org/10.1016/j.apcatb.2015.10.037>.

7. L. He, W. Zhang, S. Liu, Y. Zhao, "Three-Dimensional Porous N-Doped Graphitic Carbon Framework with Embedded CoO for Photocatalytic CO_2_ Rduction," Applied Catalysis B: Environmental 298, (2021): 120546, <https://doi.org/10.1016/j.apcatb.2021.120546>.

8. Y. Duan, Y. Wang, W. Zhang, J. Zhang, C. Ban, D. Yu, K. Zhou, J. Tang, X. Zhang, X. Han, L. Gan, X. Tao, X. Zhou, "Simultaneous CO_2_ and H_2_O Activation via Integrated Cu Single Atom and N Vacancy Dual‐Site for Enhanced CO Photo‐Production," Advanced Functional Materials 33, no. 28 (2023): 2301729, <https://doi.org/10.1002/adfm.202301729>.

9. P. Wang, Y. Dai, Z. Song, Y. Wang, J. Wei, Z. Liu, Y. Ma, F. Yang, J. Qu, S. Liu, Y. Cai, X. Yang, C. M. Li, J. Hu, "Polarized Microenvironment-Modulated Covalent Organic Frameworks with Nickel Single Atoms for Enhanced CO_2_ Photoreduction," Chemical Engineering Journal 511, (2025): 162084, <https://doi.org/10.1016/j.cej.2025.162084>.

10. H. Yang, M. Zhang, Z. Guan, J. Yang, "Cu–Fe Bimetallic MOF Enhances the Selectivity of Photocatalytic CO_2_ Reduction toward CO Production," Catalysis Science & Technology 13, no. 21 (2023): 6238, <https://doi.org/10.1039/d3cy00842h>.

11. B. Zhu, X. Hong, L. Tang, Q. Liu, H. Tang, "Enhanced Photocatalytic CO_2_ Reduction over 2D/1D BiOBr_0.5_Cl_0.5_/WO_3_ S-Scheme Heterostructure," Acta Physico Chimica Sinica 38, no. 7 (2021): 2111008, <https://doi.org/10.3866/pku.Whxb202111008>.

12. Z.-d. Lin, R.-t. Guo, Y. Yuan, X.-y. Ji, L.-f. Hong, W.-g. Pan, "Fabrication of Flower Spherical-Like Z-Scheme FeWO_4_/NiAl-LDH Photocatalysts with Excellent Activity for CO_2_ Photoreduction under Visible Light," Applied Surface Science 567, (2021): 150805, <https://doi.org/10.1016/j.apsusc.2021.150805>.

13. B. Ma, G. Chen, C. Fave, L. Chen, R. Kuriki, K. Maeda, O. Ishitani, T.-C. Lau, J. Bonin, M. Robert, "Efficient Visible-Light-Driven CO_2_ Reduction by a Cobalt Molecular Catalyst Covalently Linked to Mesoporous Carbon Nitride," Journal of the American Chemical Society 142, no. 13 (2020): 6188, <https://doi.org/10.1021/jacs.9b13930>.
